# The Development and Evaluation of a Diet Quality Index for Asian Toddlers and Its Perinatal Correlates: The GUSTO Cohort Study

**DOI:** 10.3390/nu11030535

**Published:** 2019-03-01

**Authors:** Ling-Wei Chen, Si Ming Fung, Doris Fok, Lai Peng Leong, Jia Ying Toh, Hui Xian Lim, Wei Wei Pang, Kok Hian Tan, Yap-Seng Chong, Fabian Yap, Keith M Godfrey, Yung Seng Lee, Mary Foong-Fong Chong

**Affiliations:** 1 Department of Paediatrics, Yong Loo Lin School of Medicine, National University of Singapore and National University Health System, Singapore 119228, Singapore; ling-wei.chen@ucd.ie (L.-W.C.); paeleeys@nus.edu.sg (Y.S.L.); 2 HRB Centre for Diet and Health Research, School of Public Health, Physiotherapy, and Sports Science, University College Dublin, Dublin 4, Ireland; 3 Food Science and Technology Programme, Department of Chemistry, National University of Singapore, Singapore 117543, Singapore; simingfung@gmail.com (S.M.F.); laipeng@nus.edu.sg (L.P.L.); 4 Department of Obstetrics & Gynaecology, Yong Loo Lin School of Medicine, National University of Singapore and National University Health System, Singapore 119228, Singapore; obglnld@nus.edu.sg (D.F.); obgpww@nus.edu.sg (W.W.P.); yap_seng_chong@sics.a-star.edu.sg (Y.-S.C.); 5 Singapore Institute for Clinical Sciences, Agency for Science, Technology and Research, A*STAR, Singapore 117609, Singapore; toh_jia_ying@sics.a-star.edu.sg (J.Y.T.); lim_hui_xian@sics.a-star.edu.sg (H.X.L.); 6 Department of Maternal Fetal Medicine, KK Women’s and Children’s Hospital, Singapore 229899, Singapore; tan.kok.hian@singhealth.com.sg; 7 Duke-NUS Graduate Medical School, Lee Kong Chian School of Medicine, Singapore 169857, Singapore; fabian.yap.k.p@singhealth.com.sg; 8 Department of Paediatrics Endocrinology, KK Women’s and Children’s Hospital, Singapore 229899, Singapore; 9 Medical Research Council Lifecourse Epidemiology Unit and NIHR Southampton Biomedical Research Centre, University of Southampton and University Hospital Southampton NHS Foundation Trust, Southampton SO16 6YD, UK; kmg@mrc.soton.ac.uk; 10 Khoo Teck Puat-National University Children’s Medical Institute, National University Hospital and National University Health System, Singapore 119074, Singapore; 11 Clinical Nutrition Research Centre, Singapore Institute for Clinical Sciences, A*STAR, Singapore 117599, Singapore; 12 Saw Swee Hock School of Public Health, National University of Singapore, Singapore 117549, Singapore

**Keywords:** diet quality index, toddlers, Asian, dietary guidelines, healthy diet

## Abstract

Early childhood diet may have lifelong influences on health outcomes, yet development of indices to assess diet quality is scarce in toddlers, especially in Asian countries. We aimed to develop and evaluate a Diet Quality Index (DQI) in a multi-ethnic Asian mother–offspring cohort and identify perinatal correlates of early childhood diet. Based primarily on the Singapore dietary guidelines, the DQI includes seven food components: rice, bread and alternatives; fruit; vegetables; meat and alternatives; milk and dairy products; whole grains; and foods high in sugar. The DQI was developed using parental report of Food Frequency Questionnaires (FFQ) data for 18-month-old toddlers (*n* = 561). The mean ± SD of DQI for the study toddlers was 44.2 ± 8.9 (theoretical range: 0–65). A higher DQI (better diet quality) was associated with higher intakes of several nutrients and food groups (e.g., vegetables, dietary fibre, and beta-carotene; all *p* < 0.001). Further construct validity was demonstrated by substantial agreement between the FFQ-DQI and 24-hour-recall-DQI (Intraclass-correlation-coefficient: 0.70). Independent predictors of lower DQI included higher maternal pre-pregnancy BMI [β(95% CI): −0.23(−0.39, −0.07)], Malay ethnicity [−1.88(−3.67, −0.09)], lower household income [−1.97(−3.91, −0.03)], lower education level [−2.57(−4.85, −0.28)] and never breastfeeding [−6.17(−11.06, −1.28)]. We developed a valid DQI for assessing the overall quality of the diets of Asian toddlers.

## 1. Introduction

Adequate nutrition in early childhood is essential for the achievement of each child’s human potential, with the period from birth to two years of age recognized as an especially important window for growth and development [1]. Suboptimal dietary exposures during the complementary feeding period has been shown to negatively affect future food and taste preferences [2], forming unhealthy eating habits that could translate into a higher risk of childhood obesity [3], a growing concern worldwide [4]. 

The human diet is complex with nutrient-to-nutrient interactions and a high level of correlation among intakes of certain nutrients [5]. Furthermore, the effect of an individual nutrient on health may be too small to detect [6]. Diet quality indices offer an alternative perspective of the diet, by examining the diet as a whole and measuring several aspects of dietary intake against dietary guidelines and recommendations [7]. They thus allow investigation of the determinants [8] and consequences [5] of the overall diet quality. 

Despite considerable interest in the diet quality of children, particularly those below 2 years of age [9], diet quality indices are relatively less developed in pediatric populations compared to adult populations [8]. Dietary intakes in the first two years of life are varied and less stable as toddlers are introduced to many new food and beverages during this period [10]. It may be difficult to quantify toddlers’ portion sizes as food wastage and fussy eating are prevalent among this age group. Moreover, food-based dietary guidelines with quantifiable recommendations for children below 2 years old are limited internationally [11]. Existing studies in Western countries (e.g., Netherlands [12], Puerto Rico [10] and Australia [13,14]) where diet quality indices for children have been developed and validated have demonstrated that lower index scores were generally associated with unhealthy maternal lifestyle factors such as smoking during pregnancy [12], tended to associate with higher risk of excessive weight status in toddlers [10], shorter breastfeeding duration and greater food neophobia behavior [13]. However, to our knowledge, no diet quality index has been developed for Asian toddlers in a developed country. There is a need for this research gap to be addressed as differences in Asian and Western diets [15] exist even during childhood [16]. Additionally, for the same body mass index (BMI) level, Asian populations are more susceptible to obesity-related diseases such as hypertension, cardiovascular diseases, and type 2 diabetes compared to Western populations [15,17,18]. As Singapore is a multi-ethnic country composed of major Asian ethnic groups i.e., Chinese, Malay, and Indian, it provides a unique setting for the current study.

The aim of this study is two-fold. First, we wish to develop and validate a food-based Diet Quality Index (DQI) for Asian toddlers, based primarily on local dietary recommendations. Subsequently, we aim to assess associations of maternal and toddler characteristics with the DQI.

## 2. Materials and Methods 

### 2.1. Study Population

This present study used data collected from the Growing Up in Singapore Towards healthy Outcomes (GUSTO) multi-ethnic mother–offspring cohort study [19]. Briefly, the GUSTO study is designed to investigate the influence of early life events on the risk of developing adverse health outcomes later in life. Pregnant women (*n* = 1247) within the age range of 18 to 50 years were recruited. The participants were Singapore citizens or permanent residents of Chinese, Malay or Indian descents with parents of homogenous ethnic background. They intended to deliver in National University Hospital (NUH) or KK Women’s and Children’s Hospital (KKH), to reside in Singapore for the next 5 years and agreed to donate cord, cord blood and placenta. Pregnant women who were undergoing chemotherapy, on psychotropic drugs or had type 1 diabetes mellitus were excluded. 

**Figure 1:**
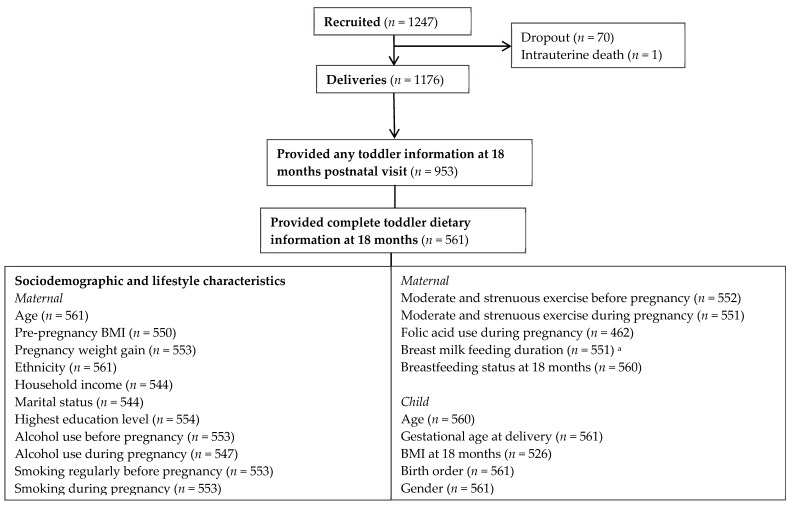
Flowchart of study participants. ^a^ Breastmilk feeding duration was assessed between delivery and 18 months postnatal visit.

A total of 1176 babies were born in the study, and 953 mother–offspring pairs completed a general questionnaire when the toddlers were 18 months old. Of these 953 pairs, 561 toddlers had complete dietary information from a food frequency questionnaire administered to mothers at the same period and were thus included in the current study. A flowchart illustrating the flow of the participants is shown in Figure 1. Compared with excluded mothers, included mothers were older, more likely to be Malay or Chinese, had higher household income and higher education level, and performed moderate and strenuous exercise before pregnancy. Other characteristics such as pre-pregnancy BMI, pregnancy weight gain, marital status, breastfeeding duration, alcohol use before and during pregnancy, moderate and strenuous exercise and folic acid use during pregnancy did not differ (Table S1). Included toddlers were slightly younger (mean age 18.3 months c.f. 18.6 months for those not included) and more likely to be first born. Gender of toddlers, gestational age at delivery, and BMI at 18 months old were comparable (Table S1). The Institutional Review Board of KKH and NUH approved the study and written informed consent was obtained from the mothers at recruitment. (Clinical trial registry: NCT01174875).

### 2.2. Data Collection

#### 2.2.1. Maternal Characteristics

Maternal ethnicity, age, marital status, education level, monthly household income, and self-reported pre-pregnancy weight were ascertained via questionnaires administered by interviewers during recruitment or during the first clinic visit. Measured maternal height (SECA stadiometer model 213) and data on folic acid supplement use during pregnancy, alcohol consumption, cigarette smoking status and moderate and strenuous exercise before and during pregnancy were gathered during a clinic visit at 26 to 28 weeks’ gestation. Pre-pregnancy BMI was derived using the formula weight (kg)/height^2^ (m^2^). To calculate pregnancy weight gain, the pre-pregnancy weight was subtracted from the weight measured (SECA weighing scale model 803) at the last antenatal clinic visit before delivery [median (interquartile range) of gestational age: 38 (37–39) weeks].

#### 2.2.2. Toddler’s Dietary Intake and Characteristics

The child’s gender, birth weight, gestational age at delivery and birth order were recorded at birth. The duration of the children receiving any breast milk was derived from face-to-face interviewer-administered feeding questionnaires during home visits at 3, 6, 9, and 12 months of age. The mothers were given a self-administered semi-quantitative Food Frequency Questionnaire (FFQ) during a home visit (15 months) and were reminded to complete the questionnaire for their toddlers 2 weeks before the 18 months clinic visit. Mothers were asked to record the dietary intakes of their toddlers for the previous month. Due to logistical constraint in visit scheduling, the time range of toddler diet captured was between 17 to 20 months of age. Pictures of standardized household measuring utensils and food portion sizes were provided to assist mothers in estimating the amount of food consumed by their toddlers. Nutrient analysis software (Dietplan, Forestfield Software, Horsham, West Sussex, UK) and a local food composition database [20] were used to perform nutrient analysis of the FFQ. Food labels or USDA national nutrient database [21] were used for food items not found in the database. The FFQ has been validated using 24-h dietary recalls in 188 toddlers. The crude correlation coefficients for energy and macronutrients (protein, fat, carbohydrate) intakes ranged from 0.42–0.52, while for micronutrients the range was 0.34–0.67 [22]. The overall mean correlation coefficient for all nutrients analysed was 0.47. The correlations improved after energy adjustment and improved further with de-attenuation to account for measurement errors (range: 0.45–0.96; mean: 0.68). These estimates were comparable with or better than previous FFQ validation studies in children aged 12-36 months, using 24-h recalls as the reference method [23]. 

At the same 18 months clinic visit, the toddlers’ weights (SECA mobile digital baby scale model 334, SECA Corp., Hamburg, Germany) and lengths (SECA mobile infant mat model 210) were measured by trained research personnel and information on main caregiver of the child was gathered through a parental-report. 

#### 2.2.3. Structure and Development of the DQI 

The DQI was developed primarily based on the Singapore dietary guidelines for toddlers aged 1 to 2 years old [24], whilst dietary guidelines from other Asian and Western countries including Hong Kong (2 to 4 years old) [25], Malaysia (3 years) [26], India (1 to 3 years) [27], Sri Lanka (1 to 2 years) [28], The Netherlands (1 to 3 years) [29], Belgium (1.5 to 3 years) [30], Germany (1 year) [31], Switzerland (1 year) [32], USA (2 years) [33], Australia (1 to 2 years) [34] were also taken into consideration. 

The final DQI consists of 7 components as illustrated in Table 1. The first 5 basic food components (total rice, bread, and alternatives, total fruit, total vegetables, total meat and alternatives and total milk and dairy products) reflect the nutritional adequacy aspect of the diet as recommended in the Singapore dietary guidelines [24]. The remaining two components reflect the quality of the diet, where introduction of whole grains into the diet and moderation in intakes of foods/beverages high in added sugar are recommended [24]. 

The scoring criteria for the seven food components, based mainly on the Singapore dietary guidelines, are listed in Table 1. We have also considered other dietary guidelines, and a comparison of these recommendations against those of Singapore is shown in Table S2. Briefly, there were differences in intake recommendations across guidelines, but these differences were relatively small (range of median % difference compared to Singapore: −9% to +39%). An exception was noted for vegetables intake, where all other guidelines unanimously recommended a higher intake (median % difference compared to Singapore = 112%). Hence, we adopted one serving as the cut off for the scoring criterion for vegetables, even though the Singapore dietary guidelines [24] recommended a 0.5 serving.

#### 2.2.4. Weighting and Scoring of DQI Components

The food items in the FFQ were first grouped according to the 5 basics food components and the weights for standard serves of the individual food items determined using the Singapore dietary guidelines [24] and a local food composition database [20]. For example, 12 fruit items e.g., bananas and papayas were grouped into the ‘total fruit’ food component. The details of classification of food items into the DQI food components and servings sizes used can be found in Supplementary Material 1. 

The basic components were scored using proportional scoring, with the ratio of actual intake to the recommended intake reflected in the score. The maximum score for each basic component was 10 points, with a higher score reflecting a higher intake of the component. Participants with 0 serving of the food component were assigned a score of 0. Details of the scoring system are available in Supplementary Material 2. For example, a child who consumed half an apple (0.5 serving) and one fifth of an orange (0.2 serving) per day had a 0.7/day (0.5 + 0.2) total serving of fruit. As the recommended serving of fruit per day is 1 serving, the child’s score would be calculated as:

$$\mathrm{Score}=\frac{\mathrm{Total}\;\mathrm{number}\;\mathrm{of}\;\mathrm{servings}\;\mathrm{consumed}}{\mathrm{Recommended}\;\mathrm{number}\;\mathrm{of}\;\mathrm{servings}\;}\times \mathrm{maximum}\;\mathrm{score}\;\mathrm{of}\;\mathrm{component}=\frac{0.7}{1}\times 10=7\;\mathrm{points}$$

The 18-month FFQ captured intakes (yes or no; but not amounts) of four food items that contained whole grains (i.e., whole meal bread, chapati, oats porridge, brown rice or porridge) (Supplementary Material 1). Participants were given 1.25 points for consuming each of the 4 food items. The maximum score for this whole grain component was 5 (rather than 10) because only variety (rather than the actual amount) of whole grains consumed was assessed.

For foods high in sugar, food items that were high in added sugar (e.g., cream cakes, sweets, carbonated soft drinks) were considered (Supplementary Material 1). The amount of sugar in these food items were obtained from a local food composition database [20] or from food labels of the food items not found in the database. The score for consumption of foods high in sugar was calculated by inverse proportional scoring, with a lower intake given a higher score closer to 10 and a higher intake given a lower score closer to 0 (see Supplementary Material 2).

The raw DQI for each toddler was derived by adding up the scores for individual components. To remove extraneous variations related to energy intake and to reduce possible measurement errors, energy adjustment of the raw DQI was performed using the nutrient residual model [35], with the toddlers’ energy intake (estimated using nutrient analysis software) and DQI included as independent and dependent variables, respectively, in linear regression. The score was then standardized to an energy intake of 845 kcal per day, which is the average energy requirement per day for 1 year old toddlers in Singapore [36]. 

### 2.3. Statistical Analysis

A summary of the sociodemographic and lifestyle characteristics of the cohort was first tabulated. Because sociodemographic and lifestyle characteristics were main exposures in this study, missing data (see Figure 1) were not imputed during analysis. 

To assess the construct validity of the DQI, the score was first categorised into tertiles. Indicators of construct validity including (i) percentage of participants who met the recommended servings/intakes of the seven food components (categorical variables); (ii) percentage of participants meeting Acceptable Macronutrient Distribution Range (AMDR) [33] for macronutrients and recommended daily allowances (RDA) [33,36] for micronutrients (categorical variables); (iii) energy adjusted dietary nutrient intakes from the FFQ (continuous variables) were summarized according to the DQI tertiles. The RDA and AMDR cutoffs were obtained from Health Promotion Board Singapore (for 1 to 2 years old) [36] and Dietary Guidelines for Americans (for 1 to 3 years old) [33]. The p-trend values for the associations between DQI and indicators of construct validity were assessed by Cochran–Mantel–Haenszel test for categorical variables and by modeling the median values of the DQI tertiles in linear regression analysis for continuous variables, with median values of DQI tertiles as the independent variable and nutrient intakes as dependent variables. 

Within a subset of 188 toddlers (but not the rest of toddlers with FFQ data) with 24-h dietary recall information, DQI from 24-h recall (DQI-24h) was calculated for each toddler and was used to further assess the construct validity of DQI from FFQ (DQI-FFQ). Mean difference between the DQI-24h and DQI-FFQ was tested using one-sample *t*-test of difference, while correlation of the two score was assessed using Pearson’s correlation coefficient. To evaluate agreement of DQI-FFQ with DQI-24h, Intraclass Correlation Coefficient (ICC) was calculated using a two-way mixed effects model assessing absolute agreement [37]. Bland–Altman analysis was also performed and potential proportional bias was assessed using linear regression analysis (with the mean difference as the dependent variable and mean score as the independent variable) [38,39]. 

Univariable linear regression models were used to assess the associations of each sociodemographic and lifestyle characteristics with the DQI. To identify independent determinants of DQI, maternal sociodemographic and lifestyle characteristics that showed *p* < 0.1 in the univariable models were included in a multivariable linear regression analysis. Furthermore, stepwise regressions with forward selection and backward elimination were also performed to assess the robustness of our results. 

All statistical analyses were performed using the statistical software IBM SPSS version 23.0 (IBM Corp., Armonk, NY, USA). Two-sided *p*-values < 0.05 were considered statistically significant. 

## 3. Results

### 3.1. Characteristics of Participants

A summary of sociodemographic and lifestyle characteristics from the included GUSTO participants can be seen in Table 2. The included cohort was made up of 57.8% Chinese, 28.0% Malay and 14.3% Indian. Most of the mothers had middle household incomes (52.6%), were married and living with their husband (95.0%), took folic acid supplement during pregnancy (73.1%), did not use alcohol before (64.2%) or during pregnancy (95.4%), did not smoke regularly before (87.0%) or during pregnancy (96.6%) and did not do moderate and strenuous exercise before (69.5%) or during pregnancy (96.6%). Approximately half of the toddlers were boys (51.5%) and first-born (47.6%). 

### 3.2. DQI Characteristics

The energy-adjusted DQI of the included toddlers ranged from 15.6 to 63.3 (theoretical range: 0–65). The DQI followed a normal distribution (histogram shown in Figure S1) with a mean ± SD of 44.2 ± 8.9. The median (IQR) score for each component (max score 10 except for whole grains) were as followed: (i) rice, bread, and alternatives: 10.0 (7.7, 10.0); (ii) fruit: 5.5 (2.8, 10.0); (iii) vegetables: 3.0 (1.5, 5.7); (iv) meat and alternatives: 10.0 (6.7, 10.0); (v) milk and dairy products: 10.0 (8.0, 10.0); (vi) whole grains: 1.3 (0, 2.5) (max score 5); (vii) foods high in sugar: 8.6 (6.6, 9.6). 

### 3.3. Construct Validity 

The construct validity of the DQI can be inferred from results presented in Table 3. Generally, those in the high DQI tertile were more likely (% range: 24.1–85.6%) to meet the recommended servings of the basic food groups, as compared with those in the low score tertile (% range: 0.0–52.4%); the p-trends were significant for all basic food groups (all *p* < 0.001) except for total milk and dairy products (*p* = 0.26). There were also increasing trends of participants meeting recommendation for whole grains intake and moderation of foods high in sugar across tertiles (both *p*-trends < 0.001). 

With regards to nutrients, those in the high score tertile tended to meet the RDA of dietary fibre, protein, calcium and vitamin A compared to the low tertile, but no significant association was observed for the AMDR of macronutrients (carbohydrates, total fat and saturated fat) and RDA of iron (Table 3). When nutrients were modeled as continuous variables, we observed that toddlers in the high score tertile had a lower proportion of energy intake from carbohydrates and a higher proportion of energy intake from protein. They also had higher intakes of dietary fibre, beta-carotene, vitamin A, cholesterol and sodium. There was no significant association observed for dietary fats (total, saturated, monounsaturated and polyunsaturated fat), iron and calcium. When DQI was modelled as a continuous variable for the abovementioned analyses, similar associations were observed. 

Within the subset of 188 toddlers with both FFQ and 24-h recall data, we observed higher DQI-24h score across tertiles of DQI-FFQ score (*p*-trend < 0.001) (Table S3). Similar to results using FFQ data, those in the high DQI-FFQ score tertile were more likely to meet recommended servings of food groups estimated using 24-h recall data (all *p* < 0.10). With regards to macronutrient intakes estimated from 24-h recall, toddlers in the high DQI-FFQ score tertile had a lower proportion of energy intake from carbohydrates and a higher proportion of energy intake from protein, whereas there was no significant association observed for total dietary fats. These results were similar to those observed using FFQ-estimated food group and nutrient intakes data.

There was a moderately strong positive correlation between energy-adjusted DQI-FFQ and DQI-24h (r = 0.66, *p* < 0.001). The ICC between DQI-FFQ and DQI-24h was 0.70. The observed correlation coefficient and ICC are comparable to results from a similar validation study involving dietary index [40]. The DQI-FFQ was higher than DQI-24h [mean difference (95% CI) = 5.73 (4.76, 6.71), *p* < 0.001], but there was no evidence of proportional bias (*p* = 0.90) (Figure S2a). When expressed as percentage difference, the 95% limits of agreement between DQI-FFQ and DQI-24h were from −20.4% to 49.7% (Figure S2b). 

### 3.4. Sociodemographic and Lifestyle Characteristics and DQI

In univariable regressions, lower DQI was associated with younger mother’s age, higher pre-pregnancy BMI, Malay ethnicity, lower household income, lower education level, smoking regularly, no moderate and strenuous exercise before pregnancy, shorter breast milk feeding duration, not breastfeeding at 18 months, higher toddlers’ BMI at 18 months old and female gender (Table 4). When these variables were included in a multivariable regression model, we found that independent predictors of a lower DQI (*p* < 0.05) included the mother’s pre-pregnancy BMI [β = −0.22 points per 1-unit increment of BMI; 95% CI: −0.38, −0.06], Malay ethnicity [β = −1.91; 95% CI: −3.70, −0.12], household income of S$2000 to 5999 [β= −1.96, 95% CI: −3.90, −0.03], education level of primary or secondary school [β = −2.53; 95% CI: −4.81, −0.25] and a shorter any breastfeeding duration (Table 4). Similar predictors were identified in stepwise regressions (Table S4). Within the subset of 188 toddlers with dietary recall information, similar trends of associations were observed between DQI-24h and (in multivariable and/or stepwise regression) Malay ethnicity, education level, (in univariable regression) household income and any breastfeeding duration (Tables S5 and S6). 

## 4. Discussion

We developed a food-based DQI that can be used to evaluate the overall diet quality of toddlers. It demonstrated adequate construct validity, with the DQI positively associated with intakes of several essential food groups, nutrients and vitamins. Furthermore, a lower DQI was also associated with sociodemographic characteristics such as higher pre-pregnancy BMI, Malay ethnicity, shorter breastfeeding duration, lower education level and lower household income. Within a subset of 188 toddlers with both FFQ and 24-h recall data, DQI-FFQ showed adequate correlation and agreement with DQI-24h.

### 4.1. Development and Construct Validity of DQI 

The components of the DQI were selected based on the recommendations provided by the Singapore dietary guidelines. To make the DQI more representative and applicable, dietary guidelines from Asian and Western countries were also considered and compared (Table S2) in deriving the scoring criteria.

Across the DQI tertiles, there was a significant increasing trend of percentage of participants meeting recommended servings of the various food components. This suggests that the standards used for scoring the food components were appropriate and could differentiate the cohort sufficiently. There was however no significant trend observed for total milk and dairy products. It has been reported that a high proportion (92%) of young children aged 3–6 years in Singapore still consume relatively high amounts of milk (mean 635 mL) daily [41]. The limited variation in milk intakes may be the reason why we did not observe differences in intakes across DQI tertiles. 

Overall, less than 30% of the participants met the recommended servings for fruit and vegetables. Although the percentages increased across DQI tertiles, they were still relatively low even in the high tertile (fruit: 52.4%; vegetables: 24.1%), suggesting that concerted effort should be made to promote fruit and vegetables intakes among Singaporean toddlers. 

Higher DQI was shown to be positively associated with higher intakes of essential nutrients and vitamins, namely, dietary fibre, beta-carotene and vitamin A, which further supports the construct validity of the DQI. Those in the highest DQI tertile also had a lower proportion of energy intake from carbohydrates, which is in line with the recommendations of dietary guidelines to limit carbohydrates, especially added sugar, in the toddlers’ diets [24]. However, the DQI was not significantly associated with calcium intake, probably due to lack of variation in milk and dairy products intakes at this age. Although DQI was not significantly associated with iron intake, there are more participants in the highest tertile that meet the RDA of iron, compared to lower tertiles. 

However, a higher DQI was associated with higher intakes of sodium and cholesterol. A possible explanation for this is that many healthy food items are rich sources of naturally occurring sodium such as cereal products, milk products and meat [42]. Furthermore, estimation of sodium intake using questionnaire is challenging and its associated measurement errors might have affected our observations [43]. Dietary cholesterol is also present in healthy foods from animal sources such as egg yolk and meat, that are valuable sources of proteins, nutrients and vitamins [44,45]. Dietary guidelines from countries such as Singapore, Korea, India and USA do not support an upper limit for dietary cholesterol but rather target saturated fat and trans-fat intake as the major determinant of blood cholesterol levels [44]. Indeed, we observed a borderline significant decreasing trend of saturated fat consumption across the DQI tertiles. 

### 4.2. Sociodemographic and Lifestyle Characteristics and DQI 

In concordance with results from other studies [46,47], we found that a lower child DQI was associated with lower maternal education level. It is possible that mothers with a lower education level are less aware or concerned about the importance of dietary quality of their toddlers [46]. Mothers with a lower education level are also more likely to be unemployed or have a low income level [46]. Indeed, we noted lower household income to be an independent predictor of lower DQI, which is in agreement with other studies in Germany [47] and the USA [48]. Households with lower incomes may not view diet quality as a priority, as healthier foods are generally more expensive [49]. It has also been shown that people with low economic status are less likely to purchase foods high in fiber, but more likely to buy high-fat and high-sugar foods, which in turn translates to a lower diet quality [50]. Moreover, our results also show that toddlers with a lower DQI were much more unlikely to meet the recommended servings of fruit and vegetables.

Other research studies have reported ethnic differences in childhood diet quality [12,51]. In our study, a lower toddler DQI was found to be associated with Malay ethnicity. This could be related to food preferences due to cultural differences [52]. It has been found, in previous studies conducted in Singapore and Malaysia, that those of Malay ethnicity tend to have less healthy diets than those of Chinese and Indian descent [49,53,54].

We observed that shorter breastfeeding duration and higher pre-pregnancy BMI were associated with lower toddler’s DQI. Following Asian BMI cut-off [55], 44.0% of our study mothers were overweight/obese (BMI ≥ 23 kg/m^2^). Results were similar when BMI was modelled as a categorical variable, i.e., toddler’s DQI of overweight/obese mothers [β (95% CI): −2.09 (−3.55, −0.64) in multivariable regression] was lower compared with normal weight mothers. Research has shown that mothers with lower pre-pregnancy BMI or mothers who breastfeed for a longer duration also tend to practice more health-conscious behaviors (e.g., consuming more servings and variety of fruits and vegetables) [56,57,58]. This suggests that the mothers’ own dietary choices may be related to the choices they make for their offspring, thus affecting the diet quality of their children [59]. However, the associations of breastfeeding and pre-pregnancy BMI with DQI could also be due to biological reasons. Dietary flavours (e.g., those from garlic, anise, carrots) can be transferred from the mothers’ diet to their child through breast milk [60]. Exposure to breast milk containing these dietary flavours has been shown to increase the toddlers’ acceptance and enjoyment of these flavors [61]. Therefore, in the case of mothers with shorter breastfeeding duration, lower toddlers’ acceptance and enjoyment of healthier foods could be one reason for a lower DQI. Maternal pre-pregnancy BMI has also been shown to cause epigenetic modifications in offspring, which may in turn alter their appetite regulation and risks of developing eating disorders [62,63]. Whether shorter breastfeeding duration and higher maternal pre-pregnancy BMI are causes or markers of lower diet quality among toddlers should be further investigated in future studies. 

Other maternal lifestyle factors such as smoking and exercise before pregnancy showed association with child’s DQI in univariable but not multivariable or stepwise regressions, indicating that other factors might have been more important. However, due to benefits associated with other pregnancy and birth outcomes it is advisable that pregnant women should avoid an unhealthy lifestyle.

### 4.3. Strengths and Limitations 

A major strength of our study is its multi-ethnic Asian population, since research studies involving diet quality indices in Asian countries are limited. We evaluated the DQI using FFQ and 24-h dietary recall within a subset of 188 toddlers and observed adequate agreement between the two methods. There was also no evidence of a proportional bias between the two methods in Bland-Altman analyses. Our DQI focused on food-based components rather than nutrients, which facilitates interpretation by the general public and application by researchers in other populations with limited food nutrient database. Furthermore, the DQI also demonstrated sufficient construct validity as it was positively associated with the intakes of essential nutrients and vitamins and was able to differentiate the diet quality of the cohort sufficiently. Potential determinants of diet quality and risk groups of poor diet quality were identified, shedding light on potential intervention targets for poor diet quality during the early childhood period.

A limitation of using diet quality index is that there is no general consensus on what makes up a healthy diet [12] and the assessment of diet quality depends on the attributes of quality selected in the individual diet score based on current dietary guidelines or current scientific literature [5]. Albeit focusing on the Singapore dietary guidelines, we also considered other dietary guidelines from Asian and Western countries in deriving the scoring criteria, potentially increasing the applicability of our DQI in other populations. However, we were limited by the availability of quantitative food-based dietary guidelines provided for young children, especially for those younger than 2 years old, a limitation that has been recognized in other research studies [11,12]. Moreover, even though the Singapore dietary guidelines provided a recommended limit for sodium intake, it was not possible to include a component on foods high in sodium in our DQI, because our FFQ was not designed to accurately measure sodium intake. Dietary variety is related to dietary quality and could be easier to compute [64]. Although our study did not focus on dietary variety, we acknowledge that a child consuming the same type of (healthy) foods everyday could be considered to have high quality diet yet score low on dietary variety. Future studies could investigate both methods together for more comprehensive child diet assessment. Another concern is the representativeness of the current study sample as only 561 (out of 953 mother–child pairs available at 18 months visit) toddlers had dietary information. We observed that included participants in this study were generally from families of higher socioeconomic status (higher household income and maternal education level) and healthier lifestyle (mothers more likely to exercise before pregnancy), as compared with not included participants. Thus, the study toddlers might have overall higher DQI compared with the rest of the cohort and general Singaporean population. Nonetheless, the internal validity of the associations with DQI for the range of sociodemographic and lifestyle characteristics included should not have been affected. Only any breastfeeding duration was considered because a very low percentage of the mothers can be considered to have exclusively breastfed their offspring. The association between duration of exclusive breastfeeding and toddler’s diet quality should be investigated in other studies. Further, we did not collect data on preconceptional folic acid supplement use, impeding investigation of the relationship between this variable and toddler’s DQI.

## 5. Conclusions

We successfully developed a DQI as an instrument to measure diet quality of Asian toddlers. The DQI showed adequate construct validity in our study population. In addition, we identified at risk groups of poor toddler’s diet quality, which may be useful in guiding future interventions by public health organizations. The DQI may also be adapted and applied to measure diet quality of young children in other populations and to further elucidate the relationship between early childhood diet and later health outcomes. 

## Figures and Tables

**Table 1:** Recommended intakes of food groups from the Singapore dietary guidelines for 1 to 2 years old and standards for scoring used for individual DQI components.

	Component	Recommended Intakes Per Day by Singapore Guidelines	Scoring Criteria Used for DQI	Maximum Score	Example of One Serving
Basic components ^a^	Total rice, bread, and alternatives	≥2–3 serves	≥2 serves	10	100 g plain rice
	Total fruit	≥0.5–1 serve	≥1 serve	10	130 g apple
	Total vegetables *	≥0.5 serve	≥1 serve	10	100 g carrot
	Total meat and alternatives	≥0.5 serve	≥0.5 serve	10	90 g chicken
	Total milk and dairy products	≥1.5 serves	≥1.5 serves	10	150 g yogurt
Additional components ^b^	Whole grains	A variety is recommended	1.25 point awarded for consumption of each food item with whole grains (4 items in total)	5	-
	Foods high in sugar	≤35 g added sugar	≤35 g added sugar	10	-

DQI, Diet Quality Index. * The scoring standard for total vegetables was adjusted from Singapore recommendation after a thorough comparison with other Asian and Western dietary guidelines (Table S2), which unanimously recommended an intake closer to 1 serving. ^a^ To calculate the score for basic components, the number of servings of each food item consumed was first determined. The number of servings of food items in each food component was then totaled. The score was then calculated by multiplying the ratio of total servings consumed to recommended servings in each component, by the maximum score. (Supplementary Material 2). ^b^ To calculate the score for foods high in sugar, the number of servings of each food item consumed and total number of servings were determined. The score was determined by deducting total number of servings from 1 and multiplying this by the maximum score. (Supplementary Material 2).

**Table 2:** Sociodemographic and lifestyle characteristics of GUSTO participants with toddler dietary information at 18 months (*n* = 561).

Maternal Characteristics	Mean ± SD or *n* (%)
Age (years)	31.3 ± 5.0
Pre-pregnancy BMI (kg/m^2^)	23.6 ± 4.6
Pregnancy weight gain (kg)	11.3 ± 4.4
Ethnicity	
Indian	80 (14.3)
Malay	157 (28.0)
Chinese	324 (57.8)
Household income category	
<S$2000	65 (11.6)
S$2000–5999	295 (52.6)
>S$6000	169 (30.1)
Missing	32 (5.7)
Marital status	
Single, not living with husband	11 (2.0)
Married, living with husband	533 (95.0)
Missing	17 (3.0)
Education level	
Primary/Secondary	149 (26.6)
Post-secondary	200 (35.7)
University and above	207 (36.9)
Missing	5 (0.9)
Alcohol use before pregnancy	
No	360 (64.2)
Yes	193 (34.4)
Missing	8 (1.4)
Alcohol use during pregnancy	
No	535 (95.4)
Yes	12 (2.1)
Missing	14 (2.5)
Smoking regularly before pregnancy	
No	488 (87.0)
Yes	65 (11.6)
Missing	8 (1.4)
Smoking during pregnancy	
No	542 (96.6)
Yes	11 (2.0)
Missing	8 (1.4)
Moderate and strenuous exercise before pregnancy	
No	390 (69.5)
Yes	162 (28.9)
Missing	9 (1.6)
Moderate and strenuous exercise during pregnancy	
No	542 (96.6)
Yes	9 (1.6)
Missing	10 (1.8)
Folic acid supplement use during pregnancy	
No	52 (9.3)
Yes	410 (73.1)
Missing	99 (17.6)
Breast milk feeding duration	
Never breastfeed	16 (2.9)
<3 months	210 (37.4)
3 to <6 months	99 (17.6)
6 to <12 months	101 (18.0)
≥12 months	125 (22.3)
Missing	10 (1.8)
Breastfeeding status at 18 months	
No	494 (88.1)
Yes	66 (11.8)
Missing	1 (0.2)
**Child characteristics**	
Age (month)	18.3 ± 0.7
Gestational age at delivery (weeks)	38.8 ± 1.4
BMI at 18 months old (kg/m^2^)	16.2 ± 1.3
Birth order	
First child	267 (47.6)
Gender	
Male	289 (51.5)
Female	272 (48.5)
Caregiver of child	
Parents	319 (56.9)
Other family members	111 (19.8)
External help	36 (6.4)
Responsibility shared	90 (16.0)
Missing	1 (0.2)

BMI, Body Mass Index.

**Table 3:** Percentages of participants meeting recommended servings of food groups or AMDR/RDA and mean nutrient intakes according to DQI tertile.

	Diet Quality Index				
	Total *n* = 561	Low Tertile *n* = 187	Middle Tertile *n* = 187	High Tertile *n* = 187	*p*-Trend ^a^
Score range	15.6–63.1	15.6–39.7	39.8–47.4	47.4–63.1	
Mean ± SD	43.2 ± 8.5	33.5 ± 4.8	43.8 ± 2.1	52.2 ± 3.7	
**% of participants meeting recommended intakes of serving/d** **ay of food groups ^b^**					
Total rice, bread, and alternatives	58.1	41.7	57.2	75.4	**<0.001**
Total fruit	28.0	9.1	22.5	52.4	**<0.001**
Total vegetable	9.8	0.0	5.3	24.1	**<0.001**
Total meat and alternatives	56.9	28.9	56.1	85.6	**<0.001**
Total milk and dairy products	52.6	52.4	47.1	58.3	0.26
Consuming whole grains ^c^	67.0	41.7	73.8	85.6	**<0.001**
Foods high in sugar ^d^	94.8	87.2	97.9	99.5	**<0.001**
**% of participants meeting AMDR/RDA of nutrients ^b^**					
Carbohydrates (AMDR: 45–65% kcal) *	83.1	84.0	82.9	82.4	0.68
Total fat (AMDR: 30–45% kcal) *	37.3	34.2	38.0	39.6	0.29
Saturated fat (AMDR: <10% kcal) *	66.7	62.0	67.4	70.6	0.08
Protein (RDA: 19 g) ^+^	98.8	96.8	99.5	100.0	**0.005**
Iron (RDA: 7 mg) ^+^	90.9	89.3	90.4	93.6	0.21
Dietary fibre (RDA: 14 g/1000 kcal) *	2.3	1.1	1.1	3.7	**0.016**
Calcium (RDA: 500 mg) ^+^	82.5	78.6	81.3	88.2	**0.021**
Vitamin A (RDA: 250 mcg) ^+^	95.7	92.0	96.3	98.9	**0.001**
**Nutrient intakes (continuous variables) ^e^**					
Carbohydrates (% of total energy)	53.5 ± 7.2	54.8 ± 7.4	53.3 ± 7.1	52.3 ± 7.1	**0.001**
Protein (% of total energy)	16.2 ± 3.5	14.6 ± 3.0	16.2 ± 3.1	17.9 ± 3.6	**<0.001**
Total fat (% of total energy)	29.7 ± 6.3	30.1 ± 6.3	29.9 ± 6.5	29.1 ± 6.1	0.12
Saturated fat (% of total energy)	7.6 ± 4.3	8.0 ± 4.8	7.5 ± 4.1	7.2 ± 4.0	0.06
Monounsaturated fat (% of total energy)	7.5 ± 3.7	7.6 ± 3.9	7.6 ± 3.7	7.3 ± 3.6	0.50
Polyunsaturated fat (% of total energy)	8.0 ± 9.8	7.9 ± 11.2	7.8 ± 9.1	8.2 ± 9.0	0.76
Iron (mg per 1000 kcal)	11.4 ± 3.3	11.7 ± 3.0	11.6 ± 3.9	11.0 ± 3.0	0.07
Dietary fibre (g per 1000kcal)	5.9 ± 3.2	4.5 ± 2.6	5.7 ± 2.4	7.5 ± 3.6	**<0.001**
Beta-carotene (mg per 1000 kcal)	2.6 ± 2.2	1.5 ± 1.6	2.5 ± 2.2	3.6 ± 2.3	**<0.001**
Cholesterol (mg per 1000 kcal)	93.1 ± 69.8	74.7 ± 74.7	92.6 ± 63.8	111.8 ± 65.8	**<0.001**
Calcium (mg per 1000 kcal)	756.4 ± 269.3	761.5 ± 287.1	756.4 ± 266.0	751.1 ± 255.3	0.71
Sodium (mg per 1000 kcal)	792.4 ± 252.4	761.3 ± 294.1	793.4 ± 236.3	822.3 ± 218.3	**0.019**
Vitamin A (mcg per 1000 kcal)	620.1 ± 255.9	558.9 ± 251.5	619.3 ± 264.2	682.2 ± 237.8	**<0.001**

DQI, Diet Quality Index; AMDR, Acceptable Macronutrient Distribution Range; RDA, Recommended Daily Allowances. ^a^ p-trend values obtained by the Cochran–Maentel–Haenzel chi-square test for categorical variables and modeling median values of the DQI tertiles in linear regression analysis for continuous variables. ^b^ Values are percentages of participants that met the recommended food group servings or AMDR/RDA within each tertile. ^c^ Since no cutoff for whole grains was provided by Health Promotion Board Singapore, values are percentages of participants that consumed at least one food item with whole grains. ^d^ For foods high in sugar, values are percentages of participants meeting recommended intake of foods and drinks high in sugar (equivalent to ≤35 g added sugar/day). ^e^ Values are the mean nutrient intake ± standard deviation within each tertile. * RDA/AMDR values obtained from Dietary Guidelines for Americans 2015–2020 (for age group 1 to 3 years old). ^+^ RDA values obtained from Health Promotion Board Singapore dietary guidelines (for age group 1 to 2 years old). Bold figures: *p*-value < 0.05

**Table 4:** Associations between sociodemographic and lifestyle factors and toddler DQI in the GUSTO cohort study (*n* = 561) ^a^.

	Univariable Model		Multivariable Model ^b^	
	β (95% CI)	*p*-Value	β (95% CI)	*p*-Value
**Maternal characteristics**				
Age (years)	0.21 (0.07, 0.35)	**0.003**	0.06 (−0.08, 0.21)	0.39
Pre-pregnancy BMI (kg/m^2^)	−0.45 (−0.60, −0.30)	**<0.001**	−0.22 (−0.38, −0.06)	**0.007**
Pregnancy weight gain (kg)	0.12 (−0.04, 0.28)	0.15	−	
Ethnicity				
Indian	0.45 (−0.05, 0.95)	0.08	−0.27 (−2.38, 1.84)	0.80
Malay	0.58 (0.20, 0.96)	**0.003**	−1.91 (−3.70, −0.12)	**0.037**
Chinese	Ref		Ref	
Household income category				
<S$2000	−6.98 (−9.33, −4.64)	**<0.001**	−1.29 (−4.18, 1.61)	0.38
S$2000–5999	−4.51 (−6.06, −2.96)	**<0.001**	−1.96 (−3.90, −0.03)	**0.047**
>S$6000	Ref		Ref	
Marital status				
Single, not living with husband	−3.22 (−8.32, 1.88)	0.22	−	
Married, living with husband	Ref			
Highest education level				
Primary/Secondary	−5.86 (−7.58, −4.13)	**<0.001**	−2.53 (−4.81, −0.25)	**0.028**
Post-secondary	−4.14 (−5.73, −2.55)	**<0.001**	−1.10 (−3.07, 0.87)	0.25
University and above	Ref		Ref	
Alcohol use before pregnancy				
No	−1.44 (−2.93, 0.05)	0.06	0.20 (−1.42, 1.81)	0.81
Yes	Ref		Ref	
Alcohol use during pregnancy				
No	−0.28 (−5.16, 4.60)	0.91	−	
Yes	Ref			
Smoking regularly before pregnancy				
No	4.32 (2.14, 6.50)	**<0.001**	0.27 (−2.13, 2.67)	0.83
Yes	Ref		Ref	
Smoking during pregnancy				
No	0.37 (−4.73, 5.47)	0.89	−	
Yes	Ref			
Moderate and strenuous exercise before pregnancy				
No	−2.43 (−3.98, −0.88)	**0.002**	−0.85 (−2.37, 0.68)	0.28
Yes	Ref		Ref	
Moderate and strenuous exercise during pregnancy				
No	1.35 (−4.28, 6.98)	0.64	−	
Yes	Ref			
Folic acid supplement use during pregnancy				
No	0.49 (−1.89, 2.86)	0.69	−	
Yes	Ref			
Breast milk feeding duration				
Never breastfeed	−8.26 (−12.37, −4.15)	**<0.001**	−6.89 (−11.98, −1.81)	**0.008**
<3 months	−7.22 (−8.97, −5.47)	**<0.001**	−5.54 (−8.02, −3.07)	**<0.001**
3 to <6 months	−2.64 (−4.72, −0.56)	**0.013**	−2.76 (−5.37, −0.15)	0.04
6 to <12 months	−2.32 (−4.39, −0.25)	**0.028**	−3.13 (−5.73, −0.53)	**0.018**
≥12 months	Ref		Ref	
Breastfeeding status at 18 months				
No	−3.94 (−6.10, −1.77)	**<0.001**	1.41 (−1.40, 4.22)	0.33
Yes	Ref		Ref	
**Child characteristics**				
Age (month)	0.68 (−0.34, 1.70)	0.19	−	
Gestational age at delivery (weeks)	0.34 (−0.16, 0.84)	0.18	−	
BMI at 18-month-old (kg/m^2^)	−0.64 (−1.18, 0.10)	**0.021**	−0.31 (−0.86, 0.23)	0.26
Birth order				
Not first child	0.21 (−1.20, 1.62)	0.77	−	
First child	Ref			
Gender				
Male	1.50 (0.09, 2.90)	**0.037**	0.93 (−0.48, 2.34)	0.20
Female	Ref		Ref	
Caregiver of child				
Parents	−1.29 (−3.28, 0.71)	0.21	0.19 (−1.87, 2.24)	0.86
Other family members	−2.18 (−4.53, 0.16)	0.07	−1.56 (−3.90, 0.78)	0.19
External help	−0.07 (−3.36, 3.22)	0.97	0.22 (−2.95, 3.40)	0.89
Responsibility shared	Ref		Ref	

BMI, Body Mass Index. ^a^ Values are beta coefficients with 95% CI from linear regression analysis. ^b^ Variables with *p* < 0.10 in univariable analysis were included in multivariable model. Bold figures: *p*-value < 0.05.

## Supplementary Materials

The following are available online at https://www.mdpi.com/2072-6643/11/3/535/s1, Figure S1: Histogram of energy-adjusted DQI based on food frequency questionnaire in the included GUSTO participants, Figure S2: Bland-Altman plot analysis of DQI-FFQ and DQI-24h, Table S1: Comparison of characteristics between included and not included participants in the current study, Table S2: Comparison of early childhood dietary guidelines,: Percentage of 188 toddlers meeting recommended servings of food groups or AMDR and mean nutrient intakes estimated from 24-h recall, according to DQI-FFQ tertile, Table S3: Percentages of 188 infants meeting recommended servings of food groups or AMDR and mean nutrient intakes estimated from 24-h recall, according to DQI-FFQ tertile, Table S4: Identification of toddler DQI-FFQ predictors in the GUSTO cohort study using stepwise regressions (*n* = 561), Table S5: Associations between sociodemographic and lifestyle factors and toddler DQI-24h recall in the subset of 188 toddlers, Table S6: Identification of toddlers DQI-24h recall predictors in the subset of 188 toddlers using stepwise regressions, Supplementary Material 1: Details of food items in DQI, Supplementary Material 2: Details of scoring a diet in DQI.
