# Supplementary material for: The Development and Evaluation of a Diet Quality Index for Asian Toddlers and Its Perinatal Correlates: The GUSTO Cohort Study
Source: Nutrients. 2019 Mar 1;11(3):535. doi: 10.3390/nu11030535 (PMC6472180; doi:10.3390/nu11030535)
Supplement: Supplementary file 1 [file nutrients-11-00535-s001.pdf]

Table S1: Comparison of characteristics between included and not included participants in the current study [a].

	Not included<br>*n* = 392	Included<br>*n* = 561	*p*-value [b]
**Maternal characteristics**			
Age (years)	30.5 ± 5.2	31.3 ± 5.0	**0.015**
Pre-pregnancy BMI (kg/m^2)	23.9 ± 4.8	23.6 ± 4.6	0.36
Pregnancy weight gain (kg)	11.2 ± 5.0	11.3 ± 4.4	0.73
Ethnicity			**<0.001**
Indian	94 (24.0)	80 (14.3)	
Malay	90 (23.0)	157 (28.0)	
Chinese	207 (52.8)	324 (57.8)	
Missing	1 (0.3)	0	
Household income category			**0.002**
< S$2000	75 (19.1)	65 (11.6)	
S$2000-5999	188 (48.0)	295 (52.6)	
> S$6000	97 (24.7)	169 (30.1)	
Missing	32 (8.2)	32 (5.7)	
Marital status			0.45
Single, not living with husband	5 (1.3)	11 (2.0)	
Married, living with husband	364 (92.9)	533 (95.0)	
Missing	23 (5.9)	17 (3.0)	
Education level			**0.041**
Primary/Secondary	129 (32.9)	149 (26.6)	
Post-secondary	134 (33.9)	200 (35.7)	
University and above	118 (30.1)	207 (36.9)	
Missing	11 (2.8)	5 (0.9)	
Alcohol use before pregnancy			0.56
No	257 (65.6)	360 (64.2)	
Yes	127 (32.4)	193 (34.4)	
Missing	8 (2.0)	8 (1.4)	
Alcohol use during pregnancy			0.35
No	367 (93.6)	535 (95.4)	
Yes	5 (1.3)	12 (2.1)	
Missing	20 (5.1)	14 (2.5)	
Smoking regularly before pregnancy			0.36
No	332 (84.7)	488 (87.0)	
Yes	5 (1.3)	65 (11.6)	
Missing	20 (5.1)	8 (1.4)	
Smoking during pregnancy			0.54
No	375 (95.7)	542 (96.6)	
Yes	10 (2.6)	11 (2.0)	
Missing	7 (1.8)	8 (1.4)	

	Not included	Included	p-value
Moderate and strenuous exercise before pregnancy			**0.001**
No	309 (78.8)	390 (69.5)	
Yes	74 (18.9)	162 (28.9)	
Missing	9 (2.3)	9 (1.6)	
Moderate and strenuous exercise during pregnancy			0.61
No	374 (95.4)	542 (96.6)	
Yes	8 (2.0)	9 (1.6)	
Missing	10 (2.6)	10 (1.8)	
Folic acid supplement use during pregnancy			0.94
No	37 (9.4)	52 (9.3)	
Yes	287 (73.2)	410 (73.1)	
Missing	68 (17.3)	99 (17.6)	
Breast milk feeding duration			0.11
Never breastfeed	24 (6.1)	16 (2.9)	
< 3 months	143 (36.5)	210 (37.4)	
3 to < 6 months	68 (17.3)	99 (17.6)	
6 to < 12 months	69 (17.6)	101 (18.0)	
≥ 12 months	72 (18.4)	125 (22.3)	
Missing	16 (4.1)	10 (1.8)	
Breastfeeding status at 18 months			
No	344 (87.8)	494 (88.1)	0.26
Yes	36 (9.2)	66 (11.8)	
Missing	12 (3.1)	1 (0.2)	
**Child characteristics**			
Age (month)	18.6 ± 1.0	18.3 ± 0.7	**<0.001**
Gestational age at delivery (weeks)	38.6 ± 1.8	38.8 ± 1.4	0.17
BMI at 18 months old (kg/m^2)	16.2 ± 1.4	16.2 ± 1.3	0.48
Birth order			**0.009**
Not first child	239 (61.0)	294 (52.4)	
First child	153 (39.0)	267 (47.6)	
Gender			0.35
Male	214 (54.6)	289 (51.5)	
Female	178 (45.4)	272 (48.5)	
Caregiver of child			0.41
Parent	216 (55.1)	319 (56.9)	
Other family members	82 (20.9)	115 (20.5)	
External help	32 (8.2)	36 (6.4)	
Responsibility shared	49 (12.5)	90 (16.0)	
Missing	13 (3.3)	1 (0.2)	

BMI, Body Mass Index

[a] Values are n (%) or mean ± standard deviation.

[b] p-values were obtained from Pearson's chi-square tests for categorical variables or independent-sample t tests for continuous variables ($p < 0.05$).

Table S2. Comparison of early childhood dietary guidelines.

Country	Singapore [1]	Netherlands [2]	Flemish [3]	Germany [4]	Switzerland [5]	USA [6]	Australia [7]	Malaysia [8]	Hong Kong [9]	India [10]	Sri Lanka [11]
For ages (y)	1 to 2	1 to 3	1.5 to 3	1	1	2	1 to 2	3	2 to 4	1 to 3	1 to 2
**1) Rice and alternatives**											
Recommended intake (g)	158	162.5	135	180	183.8	130	172.9	171.7	275	110	323.8
Difference	*NA*	*+4.5g (+2.8%)*	*-23g (-14.6%)*	*+22g (+13.9%)*	*+25.8g (+16.3%)*	*-28g (-17.7%)*	*+14.9g (+9.4%)*	*+13.7g (+8.7%)*	*+117g (+74.1%)*	*-48g (-30.4%)*	*+165.8g (+104.9%)*
**2) Fruit**											
Recommended intake (g)	108	150	150	120	120	180	150	211	108	100	252
Difference	*NA*	*+42g (+38.9%)*	*+42g (+38.9%)*	*+12g (+11.1%)*	*+12g (+11.1%)*	*+72g (+66.7%)*	*+42g (+38.9%)*	*+103g (+95.4%)*	*0 g (0 %)*	*-8g (-7.4%)*	*+126g (+133.3%)*
**3) Vegetables**											
Recommended intake (g)	50	75	75	120	120	130	187.5	106	100	100	106
Difference	*NA*	*+25g (+50%)*	*+25g (+50%)*	*+70g (+140%)*	*+70g (+140%)*	*+80g (+160%)*	*+137.5g (+275%)*	*+56g (+112%)*	*+50g (+100%)*	*+50g (+100%)*	*+56g (+112%)*
**4) Meat and alternatives**											
Recommended intake (g)	54	60	37.5	130	56.3	57	103	146.5	61.3	45	122.5
Difference	*NA*	*+6g (+11.1%)*	*-16.5g (-30.6%)*	*+76g (+141%)*	*+2.3g (+4.3%)*	*+3g (+5.6%)*	*+49g (+90.7%)*	*+92.5g (+171%)*	*+7.3g (+13.5%)*	*-9g (-16.7%)*	*+68.5g (+126.9%)*
**5) Milk and dairy products**											
Recommended intake (g)	335	310	510	300	259.6	480	228.7	280	286.7	500	500
Difference	*NA*	*-25g (-7.5%)*	*+175g (+52.2%)*	*-35g (-10.4%)*	*-75.4g (-22.5%)*	*+145g (+43.3%)*	*-106.3g (-31.7%)*	*-55g (-16.4%)*	*-48.3g (-14.4%)*	*+165g (+49.3%)*	*+165g (+49.3%)*

To standardize comparison, all recommendations were standardized to g/day intakes. Where recommendations were based on servings/day, they were first converted to g/day based on serving sizes stated within the respective guidelines. Some assumptions about the weights were required due to insufficient information provided in the guidelines. Comparisons of the Western and Asian dietary guidelines to Singapore dietary guidelines were made using absolute differences in weight (grams) and percentage differences. The absolute difference was calculated by taking the difference in recommended weights between Singapore and other dietary guidelines. The percentage difference was calculated by dividing the absolute difference over the recommended weight from Singapore guidelines and multiplying by 100. We found that while there were intake recommendation differences across guidelines, the differences were generally mixed in direction and mostly within 50% from those in Singapore guideline, thus the scoring criteria for these components were retained. The only notable difference was for vegetables intake, where all other guidelines unanimously recommended a higher intake, and with 8 out of 10 other countries recommended ≥100% more than that of Singapore guideline. Thus, we adjusted our scoring criterion for the total vegetables component in our diet quality index to 100 g (1 serving).

Supplementary Material 1: Details of food items in DQI

Components of diet quality score

Basic food groups

1. Total rice and alternatives
2. Total fruit
3. Total vegetables
4. Meat, poultry and others
5. Milk and dairy products

Additional components

Foods that are recommended

6. Whole grains

Foods that should be consumed in moderation

7. Foods high in sugar

Basic components

1. Total rice and alternatives

Food items included	Mass of an average serving size (g)	Source
White bread	60	HPB[a]
Whole meal bread	60	HPB
Bread buns with sweet filling	65	FCD[b]
Bread buns with meat filling	70	FCD
Chapati	60	HPB
Dried baby cereals total	40	HPB
Oats porridge	50	HPB
Breakfast cereals	40	HPB
Plain, cooked white rice	100	HPB
Flavoured rice	135	HPB, NAS[c]
Plain porridge, cooked with white rice	125	HPB, NAS
Flavoured porridge	125	HPB, NAS
Noodles in soup	100	HPB
Fried noodles	83	HPB, NAS
Boiled, cooked potatoes	135	HPB
Pasta, boiled	100	HPB
Boiled, cooked sweet potatoes	151	FCD
Plain crackers/ rice crackers	40	HPB

2. Total fruit

Food items included	Mass of one serving size (g)	Source
Apples and pears	130	HPB
Bananas	122	HPB
Papaya	130	HPB
Orange or citrus fruits	130	HPB

Grapes	50	HPB
Stone fruits	109	FCD
Berries	50	HPB
Watermelon	130	HPB
Raisins	40	HPB
Durian	8	NAS
Avocado	50	Research[d]
Pure fruit juice	263	HPB, NAS

3. Total vegetables

Food items included	Mass of one serving size (g)	Source
Carrots, pumpkins	100	HPB
Peas and green beans	100	HPB
Sweet corn	100	HPB
Tomato, red, green peppers	100	HPB
Dark green, leafy vegetables	100	HPB
Pale green leafy vegetables	100	HPB
Broccoli, cauliflower	100	HPB

4. Total meat and others

Food items included	Mass of one serving size (g)	Source
Chicken, duck, steamed/soup/in porridge	90	HPB
Chicken, duck, stir-fry/stewed	90	HPB
Chicken, duck, deep fried	90	HPB
Pork, beef, lamb, steamed/soup/in porridge	90	HPB
Pork, beef, lamb, stir-fry/stewed	90	HPB
Pork, beef, lamb, deep fried	90	HPB
Egg, boiled- steamed/in soup	150	HPB
Egg, fried- scrambled	150	HPB
Bean curd, tofu	170	HPB
Beans, lentils	120	HPB
Nonoily fish/ white fish- steamed/soup/in porridge	90	HPB

Nonoily fish, white fish- stir-fry/stewed	90	HPB
Nonoily fish, white fish- deep fried	90	HPB
Oily fish- steamed/in porridge	90	HPB
Oily fish- stir-fry	90	HPB
Oily fish- deep fried	90	HPB

5. Total milk and dairy products

Food items included	Mass of one serving size (g)	Source
Infant formula	500	HPB
Full cream milk/fresh milk/flavoured milk	516	HPB
Low fat milk/fresh milk/flavoured milk	518	HPB
Yogurt	150	FCD
Cheese	20	FCD
Breast milk (volume expressed or volume imputed from partial or full lactation)	500	HPB

Additional components

6. Whole grains (recommended by Singapore dietary guidelines)

Food items included
Whole meal bread
Chapati
Oats porridge
Brown rice or porridge

7. Foods high in sugar (consume in moderation)

Food items included	Weight of food item containing 35g sugar (g)
Sponge cakes / steamed cakes	136
Cream cakes	98
Chocolate	44
Sweets	44
Ice-cream	160

Malted drinks	560
Fruit drinks and juices	332
Carbonated soft drinks	330
Non-carbonated soft drinks	613
Soya milk	465
Traditional drinks	380
Jams/ Honey	52
Peanut Butter	317
Kaya	81

Description of sources

[a]HPB: Singapore dietary guidelines for 1 to 2 years old provided by health promotion Board (HPB) [12]

[b]FCD: local food composition database [13]

[c]NAS: nutrient analysis software (Dietplan, Forestfield software, UK)

[d]Research: an average of 3 published serving sizes of avocado [14–16]

Supplementary Material 2: Details of scoring a diet in DQI

Basic components

The weight of food item consumed by subjects per day was calculated from the FFQ, which was then converted to number of servings using weights for a standard serve. The number of servings for each food item consumed by the participant and subsequently, each food component was calculated and the score obtained by the participant was determined.

This is explained using the example of person XX who had half an apple (65g apple) and one fifth or an orange (26g) in 1 day; Standard serving sizes of apple and orange are both 130g. The recommended serving of fruit a day is 1 serving.

Step 1: Determination of number of servings of each food item consumed

Number of servings of apple = $\frac{weight\ of\ intake\ amount}{weight\ of\ 1\ serving}$

$= \frac{65}{130}$ = 0.5 servings

Number of servings of orange = $\frac{26}{130}$ = 0.2 servings

Step 2: Add up the number of servings of food items in the food component to get the total number of servings in each food component

Number of servings of fruit consumed = 0.5 + 0.2 = 0.7 servings

Step 3: Determination of score

Score = $\frac{Total\ number\ of\ servings\ consumed}{Recommended\ number\ of\ servings}$ x maximum score of component

$= \frac{0.7}{1}\ x\ 10$ = 7 points

Additional components- foods high in sugar

This is explained using the example of person XY who had 10g of chocolate and 40g of ice-cream in 1 day; 44.09g and 160.12g of chocolate and ice-cream respectively contains 35g of sugar and is taken to be 1 serving.

(35g sugar is the sugar intake limit recommended by Singapore dietary guidelines[1]. A lower intake of sugar is recommended.)

Step 1: Determination of number of servings of each food item consumed

Number of servings of chocolate = $\frac{10}{44.09}$ = 0.226 servings

Number of servings of ice cream = $\frac{40}{160.12}$ = 0.249 servings

Step 2: Add up the number of servings of food items in the food component

Number of servings of foods high in sugar = 0.226 + 0.249 = 0.475 servings

Step 3: Determination of score

Score = (1 – Total number of servings) x 10 = (1 – 0.475) x 10 = 5.25

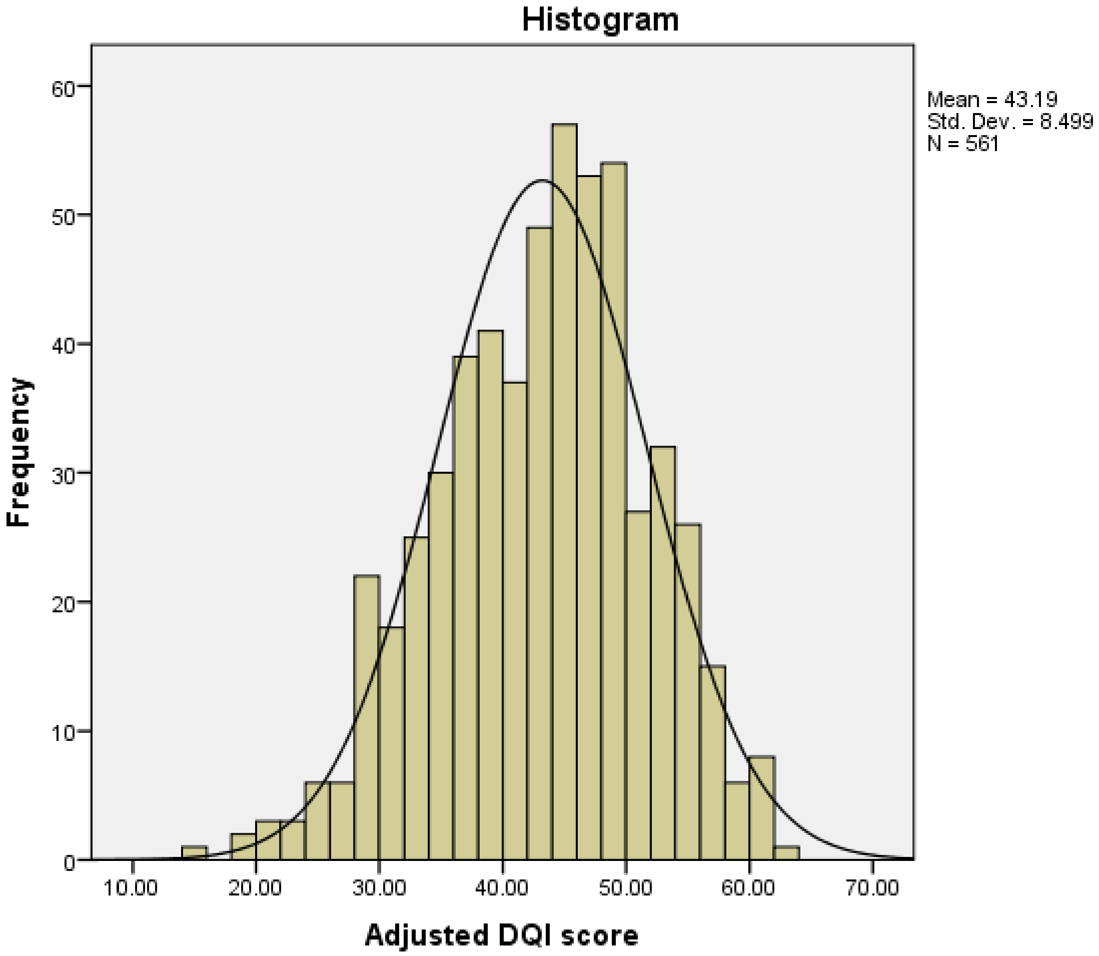

Figure S1: Histogram of energy-adjusted DQI based on food frequency questionnaire in the included GUSTO participants. DQI, Diet Quality Index.

Table S3. Percentages of 188 infants meeting recommended servings of food groups or AMDR and mean nutrient intakes estimated from 24-h recall, according to DQI-FFQ tertile.

	DQI-FFQ				
	**Total** n = 188	**Low tertile** n = 65	**Middle tertile** n = 63	**High tertile** n = 60	**p-trend**[a]
Score range	22.1 – 63.1	22.1 – 39.6	39.9 – 47.3	47.6 – 63.1	
**DQI - 24h recall**[b]					
Mean ± SD	37.4 ± 8.3	31.2 ± 5.1	38.1 ± 6.2	43.3 ± 8.4	**<0.001**
Range	19.3 – 62.7	19.3 – 44.8	24.1 – 53.3	22.1 – 62.7	
**% of participants meeting recommended intakes of servings/d of food groups**[c]					
Total rice, bread and alternatives	23.4	20.0	17.5	33.3	0.085
Total fruit	12.2	1.5	14.3	21.7	**0.001**
Total vegetable	6.4	0.0	4.8	15.0	**0.001**
Total meat and alternatives	37.8	18.5	41.3	55.0	**<0.001**
Total milk and dairy products	43.6	35.4	41.3	55.0	**0.028**
Consuming whole grains[d]	27.7	12.3	23.8	48.3	**<0.001**
Foods high in sugar[e]	98.9	96.9	100.0	100.0	0.090
**% of participants meeting AMDR/RDA of nutrients**[c]					
Carbohydrates (AMDR: 45-65% kcal)*	70.7	63.1	79.4	70.0	0.373
Total fat (AMDR: 30-45% kcal)*	38.3	32.3	38.1	45.0	0.146
Protein (RDA: 19g)+	96.3	92.3	98.4	98.3	0.072
**Nutrient intakes (continuous variables)**[f]					
Carbohydrates (% of total energy)	58.0 ± 8.2	60.0 ± 8.8	57.1 ± 7.3	56.8 ± 8.1	**0.025**
Protein (% of total energy)	14.6 ± 2.9	13.7 ± 2.6	14.4 ± 2.8	15.9 ± 2.8	**<0.001**
Total fat (% of total energy)	29.8 ± 7.1	28.9 ± 7.4	30.8 ± 7.0	29.6 ± 6.7	0.491

DQI, Diet Quality Index; FFQ, Food Frequency Questionnaire; SD, Standard Deviation, AMDR, Acceptable Macronutrient Distribution Range; RDA, Recommended Daily Allowances

[a] p-trend values obtained by the Cochran-Maentel-Haenzel chi-square test for categorical variables and modeling median values of the DQI-FFQ tertiles in linear regression analysis for continuous variables.

[b] DQI calculated from a subset of 188 infants using 24-h dietary recalls.

[c] Values are percentages of participants that met the recommended food group servings or AMDR/RDA (data from 24-h recall) across DQI-FFQ tertiles.

[d] Since no cutoff for whole grains was provided by Health Promotion Board Singapore, values are percentages of participants that consumed at least one food item with whole grains.

[e] For foods high in sugar, values are percentages of participants meeting recommended intake of foods and drinks high in sugar (equivalent to ≤ 35g added sugar/d).

[f] Values are the mean nutrient intake ± standard deviation (data from 24-h recall) across DQI-FFQ tertiles.

* RDA/AMDR values obtained from Dietary Guidelines for Americans 2015-2020 (for age group 1 to 3 years old).

+ RDA values obtained from Health Promotion Board Singapore dietary guidelines (for age group 1 to 2 years old).

(a) Difference of DQI-FFQ and DQI-24h against mean scores

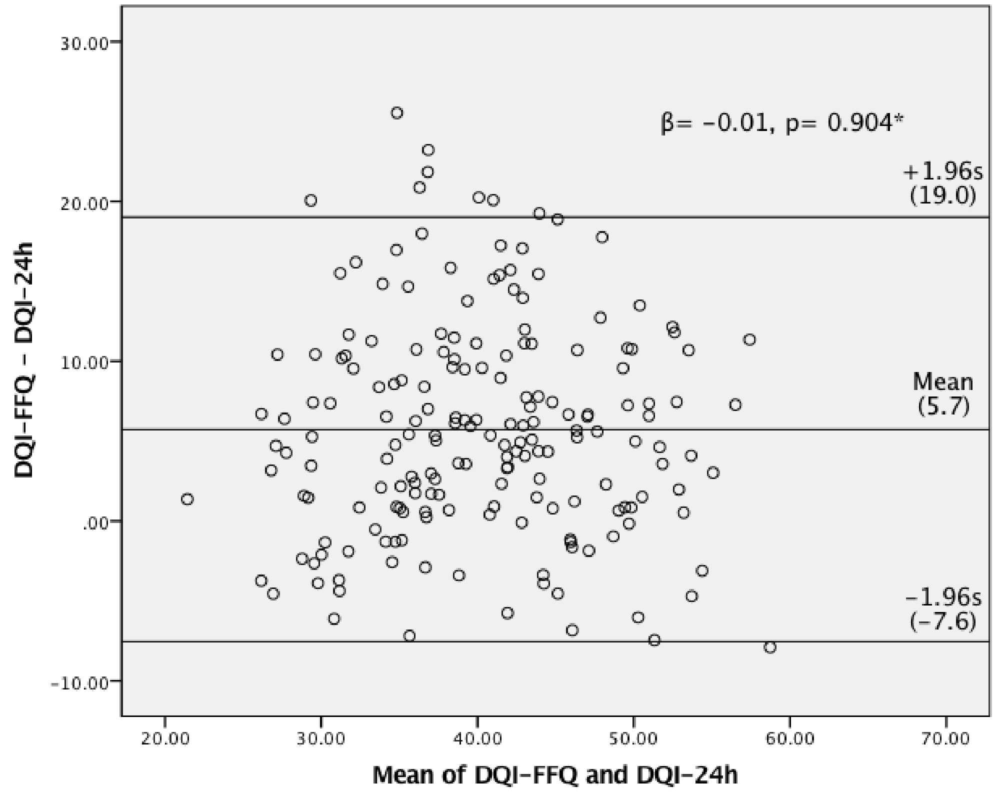

(b) Percentage difference between DQI-FFQ and DQI-24h against mean scores

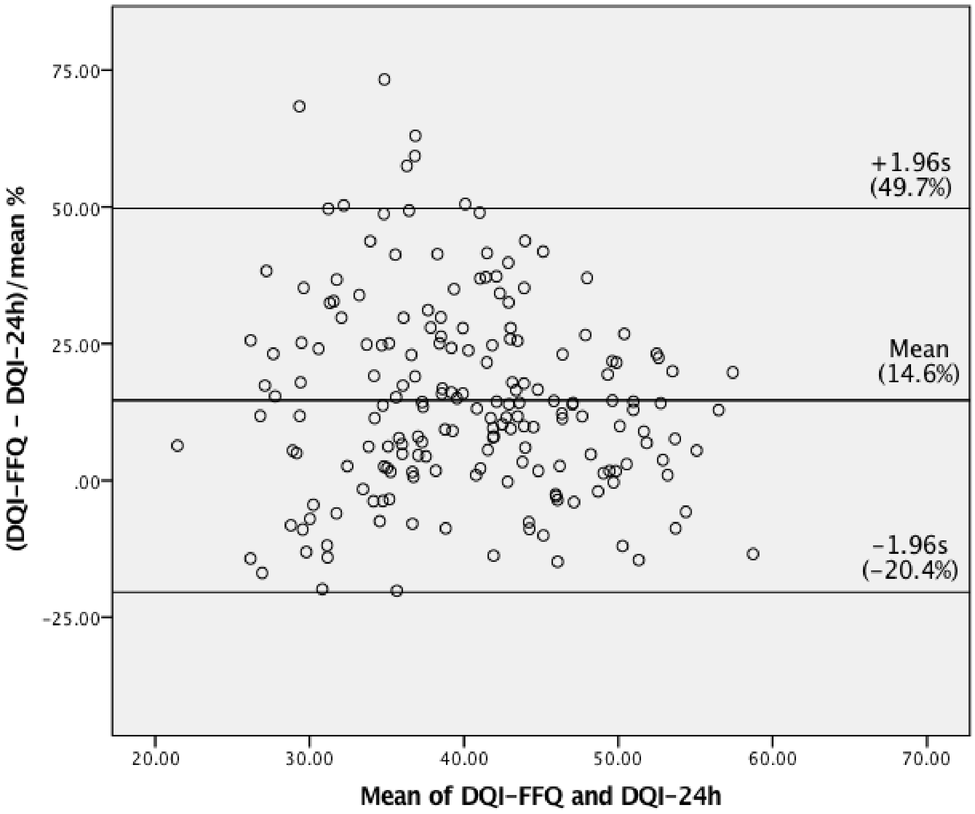

Figure S2. Bland-Altman plot analysis of DQI-FFQ and DQI-24h. * Regression coefficient and statistical significance from linear regression of the difference between methods (dependent variable) against mean (independent variable).

Table S4. Identification of infant DQI-FFQ predictors in the GUSTO cohort study using stepwise regressions (n = 561).

	Forward selection model		Backward elimination model	
	β (95% CI)	*p*-value	β (95% CI)	*p*-value
**Maternal characteristics**				
Age (years)	–		–	
Pre-pregnancy BMI (kg/m²)	-0.25 (-0.40, -0.09)	**0.002**	-0.24 (-0.39, -0.08)	**0.003**
Pregnancy weight gain (kg)	–		–	
Ethnicity				
Indian	–		–	
Malay	-2.04 (-3.68, -0.40)	**0.015**	-2.00 (-3.62, -0.37)	**0.016**
Chinese	Ref		Ref	
Household income category				
< $2000	–		–	
$2000-5999			-1.37 (-2.88, 0.15)	0.08
> $6000			Ref	
Marital status				
Single, not living with husband	–		–	
Married, living with husband				
Highest education level				
Primary/Secondary	-3.50 (-5.43, -1.58)	**<0.001**	-2.90 (-4.90, -0.91)	**0.004**
Post-secondary	-2.22 (-3.94, -0.49)	**0.012**	-1.66 (-3.49, 0.17)	0.08
University and above	Ref		Ref	
Alcohol use before pregnancy				
No	–		–	
Yes				
Alcohol use during pregnancy				
No	–		–	
Yes				
Smoking regularly before pregnancy				
No	–		–	
Yes				
Smoking during pregnancy				
No	–		–	
Yes				
Moderate and strenuous exercise before pregnancy				
No	–		–	
Yes				
Moderate and strenuous exercise during pregnancy				
No	–		–	
Yes				

	Forward selection model		Backward elimination model	
	β value (95% CI)	*p*-value	β value (95% CI)	*p*-value
Folic acid supplement use during pregnancy				
No	–		–	
Yes				
Breast milk feeding duration				
Never breastfeed	-5.00 (-9.67, -0.32)	**0.036**	-6.93 (-11.72, -2.14)	**0.005**

< 3 months	-3.93 (-5.49, -2.37)	**<0.001**	-5.56 (-7.49, -3.64)	**<0.001**
3 to < 6 months	–		-2.39 (-4.54, -0.23)	**0.030**
6 to < 12 months	–		-2.89 (-5.05, -0.74)	**0.009**
≥12 months	Ref		Ref	
Breastfeeding status at 18 months				
No	–		–	
Yes				
**Child characteristics**				
Age (month)	–		–	
Gestational age at delivery (weeks)	–		–	
BMI at 18 months old (kg/m^2)	–		–	
Birth order				
Not first child	–		–	
First child				
Gender				
Male	–		–	
Female				
Caregiver of child				
Parents	–		–	
Other family members				
External help				
Responsibility shared				

DQI, Diet Quality Index; FFQ, food frequency questionnaire; BMI, Body Mass Index.

Table S5. Associations between sociodemographic and lifestyle factors and infant DQI-24h recall in the subset of 188 infants[a.]

	**Univariable model**		**Multivariable model** [b]	
	β (95% CI)	p-value	β (95% CI)	p-value
**Maternal characteristics**				
Age (years)	0.42 (0.20, 0.64)	**<0.001**	0.23 (0.01, 0.45)	**0.046**
Pre-pregnancy BMI (kg/m^2)	-0.42 (-0.67, -0.16)	**0.001**	-0.15 (-0.40, 0.11)	0.26
Pregnancy weight gain (kg)	0.14 (-0.13, 0.40)	0.15	–	
Ethnicity				
Indian	-4.26 (-7.49, -1.04)	**0.010**	-3.66 (-7.18, -0.13)	**0.042**
Malay	-8.23 (-10.72, -5.74)	**<0.001**	-5.91 (-8.79, -3.03)	**<0.001**
Chinese	Ref		Ref	
Household income category				
< S$2000	-6.47 (-10.50, -2.45)	**0.002**	0.99 (-3.66, 5.64)	0.68
S$2000-5999	-4.55 (-7.43, -1.67)	**0.002**	0.26 (-3.12, 3.63)	0.88
> S$6000	Ref		Ref	
Marital status				
Single, not living with husband	-9.81 (-21.33, 1.72)	0.10	–	
Married, living with husband	Ref			
Highest education level				
Primary/Secondary	-6.21 (-9.07, -3.35)	**<0.001**	-3.63 (-7.42, 0.15)	0.06
Post-secondary	-4.69 (-7.41, -1.97)	**0.001**	-2.10 (-5.61, 1.41)	0.24
University and above	Ref		Ref	
Alcohol use before pregnancy				
No	-1.10 (-3.80, 1.61)	0.42	–	
Yes	Ref			
Alcohol use during pregnancy				

No	-3.02 (-19.54, 13.50)	0.72	–	
Yes	Ref			
Smoking regularly before pregnancy				
No	5.91 (2.27, 9.56)	**0.002**	1.97 (-1.91, 5.85)	0.32
Yes	Ref		Ref	
Smoking during pregnancy				
No	2.59 (-4.25, 9.44)	0.46	–	
Yes	Ref			
Moderate and strenuous exercise before pregnancy				
No	-2.35 (-5.12, 0.42)	0.10	–	
Yes	Ref			
Moderate and strenuous exercise during pregnancy				
No	-7.36 (-16.89, 2.17)	0.13	–	
Yes	Ref			

	**Univariable model**		**Multivariable model**	
	β (95% CI) [a]	p-value	β (95% CI) [b]	p-value
Folic acid supplement use during pregnancy				
No	-0.05 (-3.34, 3.24)	0.98	–	
Yes	Ref			
Breast milk feeding duration				
Never breastfeed	-4.68 (-10.80, 1.43)	0.13	-5.08 (-11.29, 1.14)	0.11
<3 months	-4.56 (-7.56, -1.54)	**0.003**	-2.26 (-5.43, 0.91)	0.16
3 to <6 months	-0.68 (-4.27, -2.91)	0.71	-2.15 (-5.60, 1.30)	0.22
6 to <12 months	-2.32 (-4.39, -0.25)	0.83	-2.29 (-6.01, 1.43)	0.23
≥12 months	Ref		Ref	
Breastfeeding status at 18 months				
No	-1.77 (-5.12, 1.58)	0.30	–	
Yes	Ref			
**Child characteristics**				
Age (month)	1.21 (-1.57, 3.98)	0.39	–	
Gestational age at delivery (weeks)	0.12 (-0.83, 1.07)	0.80	–	
BMI at 18-month-old (kg/m^2)	0.03 (-0.02, 0.08)	0.28	–	
Birth order				
Not first child	0.79 (-1.62, 3.21)	0.52	–	
First child	Ref			
Gender				
Male	2.39 (0.02, 4.77)	**0.048**	1.76 (-0.54, 4.06)	0.13
Female	Ref		Ref	
Caregiver of child				
Parents	-1.49 (-4.97, 1.99)	0.40	–	
Other family members	-1.98 (-6.41, 2.45)	0.38		
External help	1.47 (-5.14, 8.07)	0.66		
Responsibility shared	Ref			

BMI, Body Mass Index

[a] Values are beta coefficients with 95% CI from linear regression analysis.

[b] Variables with p <0.10 in univariable analysis were included in multivariable model.

Table S6. Identification of infant DQI-24h recall predictors in the subset of 188 infants using stepwise regressions.

	Forward selection model		Backward elimination model	
	β (95% CI)	p-value	β (95% CI)	p-value
**Maternal characteristics**				
Age (years)	0.31 (0.10, 0.52)	**0.005**	0.28 (0.06, 0.49)	**0.011**
Pre-pregnancy BMI (kg/m^2)	–		–	
Pregnancy weight gain (kg)	–		–	
Ethnicity				
Indian	–		-3.57 (-6.99, -0.16)	**0.040**
Malay	-6.30 (-8.89, -3.72)	**<0.001**	-6.29 (-9.02, -3.56)	**<0.001**
Chinese	Ref		Ref	
Household income category				
< $2000	–		–	
$2000-5999				
> $6000				
Marital status				
Single, not living with husband	–		–	
Married, living with husband				
Highest education level				
Primary/Secondary	-2.85 (-5.32, -0.37)	**0.024**	-4.21 (-7.15, -1.26)	**0.005**
Post-secondary	–		-2.47 (-5.33, 0.39)	0.09
University and above	Ref		Ref	
Alcohol use before pregnancy				
No	–		–	
Yes				
Alcohol use during pregnancy				
No	–		–	
Yes				
Smoking regularly before pregnancy				
No	–		–	
Yes				
Smoking during pregnancy				
No	–		–	
Yes				
Moderate and strenuous exercise before pregnancy				
No	–		–	
Yes				
Moderate and strenuous exercise during pregnancy				
No	–		–	
Yes				

	Forward selection model		Backward elimination model	
	β value (95% CI)	p-value	β value (95% CI)	p-value
Folic acid supplement use during pregnancy				
No	–		–	
Yes				
Breast milk feeding duration				
Never breastfeed	–		–	
< 3 months				

3 to < 6 months				
6 to < 12 months				
≥12 months				
Breastfeeding status at 18 months				
No	–		–	
Yes				
**Child characteristics**				
Age (month)	–		–	
Gestational age at delivery (weeks)	–		–	
BMI at 18 months old (kg/m^2)	–		–	
Birth order				
Not first child	–		–	
First child				
Gender				
Male	-2.44 (-4.68, -0.21)	**0.033**	-1.97 (-4.23, 0.28)	0.09
Female	Ref		Ref	
Caregiver of child				
Parents	–		–	
Other family members				
External help				
Responsibility shared				

DQI, Diet Quality Index; BMI, Body Mass Index.

References

1. HPB. A Healthy Food Foundation - for Kids and Teens [Internet]. Health Promotion Board. 2015. Available from: https://www.healthhub.sg/live-healthy/578/A Healthy Food Foundation - for Kids and Teens
2. Netherlands Nutrition Centre (Voedingscentrum). Dutch Food-based Dietary Guidelines (Richtlijnen voedselkeuze). 2011.
3. Flemish Institute for Health Promotion and Disease Prevention. De actieve voedingsdriehoek [The Active Food Guide Pyramid]. 2012.
4. Kersting M HA. Ernährung bei Kleinkindern: Empfehlungen und Ernahrungspräxis. (Nutrition in infants: recommendations and nutritional practice). 14. 2012. p. 24–9.
5. Schweizerische Gesellschaft für Ernährung. Ernährung von Kindern (Nutrition of children). 2011. p. 6.
6. USDA. 2015 – 2020 Dietary Guidelines for Americans. 2015 – 2020 Diet Guidel Am (8th Ed. 2015;18.
7. National Medical Health and Research Council. Australian Dietary Guidelines. National Health and Medical Research Council; 2013.
8. MOH. Malaysian Dietary Guidelines for Children and Adolescents. 2013;1–74.
9. Department of Health. Nutrition Guidelines for Children Aged 2 to 6 (Hong Kong). 2014. p. 1–150.
10. National Institute of Nutrition. Dietary Guidelines for Indians - A Manual. 2011. p. 139.
11. Nutrition Division Ministry of Health. Food Based Dietary Guidelines for Sri Lankans. 2011.
12. HPB. Healthier Choice Symbol Programme [Internet]. 2015 [cited 2015 Oct 27]. Available from: http://www.hpb.gov.sg/HOPPortal/health-article/2780
13. Singapore HPB. Energy and Nutrient Composition of food. 2011.
14. Comerford K, Ayoob K, Murray R, Atkinson S. The Role of Avocados in Complementary and Transitional Feeding. Nutrients. 2016 May 21;8(5):316.
15. DAA. Avocados | Dietitians Association of Australia [Internet]. 2016 [cited 2016 Dec 26]. Available from: http://daa.asn.au/for-the-public/smart-eating-for-you/nutrition-a-

z/avocados/

16. NHS. Rough guide - Fruit & vegetable portion sizes [Internet]. 2015 [cited 2016 Dec 26]. p. 1–9. Available from: https://www.nhs.uk/Livewell/5ADAY/Documents/Downloads/5ADAY_portion_guide.pdf
